# Significance of peripheral blood indexes in differential diagnoses of SARS-CoV-2 and New Bunia virus

**DOI:** 10.1038/s41598-021-93519-8

**Published:** 2021-07-08

**Authors:** Wentao He, Xiaoyi Liu

**Affiliations:** 1 Department of Clinical Laboratory, The First Affiliated Hospital of Anhui Medical University, Jixi Road 218, Shushan District, Hefei, 230022 Anhui People’s Republic of China; 2 Department of Clinical Nutrition, The First Affiliated Hospital of USTC, Division of Life Sciences and Medicine, University of Science and Technology of China, Hefei, 230036 Anhui China

**Keywords:** Biomarkers, Outcomes research, Infectious diseases, Respiratory tract diseases

## Abstract

We aimed to provide a laboratory basis for differential diagnosis of COVID-19 and severe fever with thrombocytopenia syndrome (SFTS). Clinical data were collected from 32 COVID-19 patients (2019-nCoV group), 31 SFTS patients (SFTS group) and 30 healthy controls (control group). For each group of hospitalized patients, a retrospective analysis was performed on specific indices, including cytokines, T-lymphocyte subsets, routine blood parameters, C-reactive protein (CRP) and procalcitonin (PCT), and receiver operating characteristic (ROC) curves for the indices revealed the differences among groups. Compared with the 2019-nCoV group, the SFTS group had a significantly and greatly decreased counts of WBC, absolute lymphocyte, PLT and absolute CD4^+^ T lymphocyte (*P* < 0.05); the IL-6, TNF-α, D-D and PCT levels of the SFTS group were higher than those of the 2019-nCoV group (*P* < 0.05). Compared with those of the SFTS group, the CRP and FIB levels of the 2019-nCoV group were greatly increased (*P* < 0.05). The ROC curves showed that area under the curves (AUCs) for FIB, PLT and TNF-α were greater than 0.85, demonstrating high diagnostic value. At the initial stage of SARS-CoV-2 or SFTS virus infection, PLT, FIB and TNF-α have definitive clinical value for the early and differential diagnosis of these two infections.

## Introduction

Since the end of 2019, when the Corona Virus Disease 2019 (COVID-19) caused by SARS-CoV-2 was first reported in Wuhan, the pandemic has spread worldwide^1,2^.

As of 23 November 2020, 58,892,589 confirmed COVID-19 cases and 1,388,240 deaths had been reported and the World Health Organization (WHO) had indicated a very high global risk level for this disease^3^.

SARS-CoV-2 is a single-stranded RNA virus belonging to the β-genus of the Coronaviridae family^4^. The pathophysiological mechanism of SARS-CoV-2 is still not yet fully understood. However, according to the literature, the human angiotensin-converting enzyme 2 (ACE2) receptor may mediate the process of SARS-CoV-2 recognition and cell infection. In fact, human lung tissue contains a large number of ACE2 receptors^5^.

Relevant studies also have shown that the impairment of cellular immunity, the increase in proinflammatory cytokines and the inflammatory cascade effect, i.e., the cytokine storm theory proposed by academic Li Lanjuan may be involved in its pathogenesis^6–9^. The initial symptoms of COVID-19 are not typical. Fever is the main manifestation, while a few cases have shown other clinical symptoms, such as fatigue, sore throat, myalgia and diarrhea^8^. However, the peak season for severe fever with thrombocytopenia syndrome (SFTS) infections occurs each year, from April to August^10,11^. In fact, the New Bunia virus, a single-stranded negative-sense RNA virus, has been named the severe fever with thrombocytopenia syndrome virus, or SFTSV. The pathogenic mechanism of SFTSV has not yet been completely understood. However, studies have shown that the SFTSV envelope glycoprotein Gn/Gc plays an important role in mediating virus invasion into cells^12^.

SFTS caused by SFTSV infection is an acute infectious disease without clinical specialty and may easily be misdiagnosed^13^. Its main clinical manifestations include fever, a reduction in platelets and leukopenia. Most of those infected also suffer from other clinical symptoms, such as fatigue, gastrointestinal symptoms, muscle soreness and lymphadenectasis^14^. Studies have shown that the impairment of adaptive immune function and abnormal induction of inflammatory cytokines also play a significant role in the SFTS pathogenic process^15^.

Although two viruses with different transmission routes (tick, infected material contact vs. respiratory tract), contamination areas-epidemiology (rural vs. crowded areas) and clinic (main hematological system vs. major respiratory system) were compared, the early laboratory indices of two viral infections has not yet been compared.

To date, positive SARS-CoV-2 nucleic acid testing results are still the basis on which COVID-19 cases are confirmed^16^. While the SARS-CoV-2 nucleic acid test has strict requirements for laboratory testing conditions, false negative results and missed diagnoses may exist. Radiological examinations (CT) as a second option in the evaluation of PCR-negative COVID-19 patients may offer more valuable results^16^. However, many hospitals do not have expensive ct scanning equipment in some developing countries.

In this study, to enhance the understanding of COVID-19 and SFTS and to provide laboratory evidence for early differential diagnosis and treatment of COVID-19 and SFTS, a retrospective analysis was carried out on a series of initial onset indices for 32 confirmed COVID-19 cases and 31 SFTS cases. These indices included interleukin-6 (IL-6), tumor necrosis factor alpha (TNF-α), C-reactive protein (CRP), procalcitonin (PCT), white blood cell (WBC) count, lymphocyte (LYMPH) count, platelet (PLT) count, fibrinogen (FIB), d-dimer (D-D), CD4^+^ T lymphocyte (CD4^+^ T) count, CD8^+^ T lymphocyte (CD8^+^ T) count and CD4^+^ T/CD8^+^ T ratio (CD4^+^ T/CD8^+^ T).

## Materials and methods

### General information

The cases examined in this study included 32 confirmed COVID-19 cases at the First Affiliated Hospital of Anhui Medical University from January 27, 2020, to February 21, 2020, and 31 SFTS cases treated at the First Affiliated Hospital of Anhui Medical University from July 1, 2019, to April 26, 2020. The two groups were named the 2019-nCoV group and the SFTS group, respectively. In addition, 30 healthy people who had recently undergone physical examinations were selected to form the control group. The inclusion criteria were as follows: (1) 2019-nCoV group: patients with positive SARS-CoV-2 nucleic acid testing results; (2) SFTS group: patients with one of the following pathogenic findings: positive serum SFTSV nucleic acid testing results, positive serum immunological testing results, or positive serum virus isolation and culture testing results. The exclusion criteria were as follows: cases with obvious bacterial infections such as tonsil inflammation, bacterial pneumonia and urethritis. Additionally, members from the control group were required to be negative for SARS-CoV-2 by the nucleic acid test.

### Methods

Within 1 day after being hospitalized, patients had their blood drawn to test the following: CD4^+^ T cell count, CD8^+^ T cell count and CD4^+^ T/CD8^+^ T ratio, using the BD FACSCanto Plus flow cytometer (BD, USA); routine blood tests, using the XN-9000 automatic hematology analyzer (Sysmex, Japan); FIB and D-D, using the STAGO automatic coagulometer (Stago, France); CRP, using the c8000 automatic biochemical analysis system (Abbott, USA); PCT, using the mini VIDAS(Merrier, USA); and IL-6 and TNF-α, using the Roche c6000 automatic immune analyzer(Roche, USA).

### Statistical methods

SPSS 17.0 statistical software was utilized for data analysis. The measurement data conforming to a normal distribution are represented by X ± S, and the measurement data conforming to a non-normal distribution are represented by M (P25–P75). For variables with normal distribution, a one-way ANOVA was used for multigroup analyses, and the LSD method was used to conduct pairwise analyses. For variables with non-normal distribution, Kruskal–Wallis H tests were performed for multigroup analyses, and Mann–Whitney U tests were performed for pairwise analyses. Statistical significance is indicated when *P* < 0.05. The receiver operating characteristic curve (ROC curve) was prepared, and the area under the ROC curve (AUC) was calculated to evaluate diagnostic efficacy.

### Ethical approval

All procedures performed in studies involving human participants were in accordance with the ethical standards of the institutional (The First Affiliated Hospital of Anhui Medical University) and/or national research committee and with the 1964 Helsinki declaration and its later amendments or comparable ethical standards.

### Informed consent

Informed consent was obtained from all individual participants included in the study.

### Statement

All experimental protocols were approved by the Ethics Committee of The First Affiliated Hospital of Anhui Medical University, and all methods were carried out in accordance with relevant guidelines and regulations.

## Results

### General data of patients

The patients from the 2019-nCoV group were between 21 and 71 years old, with an average age of 43.5 ± 12.4 years, and male patients accounted for 56.3%. The patients from the SFTS group were between 30 and 72 years old, with an average age of 49.3 ± 11.2 years, and male patients accounted for 54.8%. The members of the control group were between 29 and 71 years old, with an average age of 45.9 ± 11.2, and male patients accounted for 53.3%. The comparison of the basic data among the 3 groups, including age and sex, showed no significant difference (*P* < 0.05), which means that they were comparable.

### Comparison of WBC, PLT, LYMPH, CD4^+^ T, CD8^+^ T, CD4^+^ T/CD8^+^ T among the 3 groups

**Figure 1:**
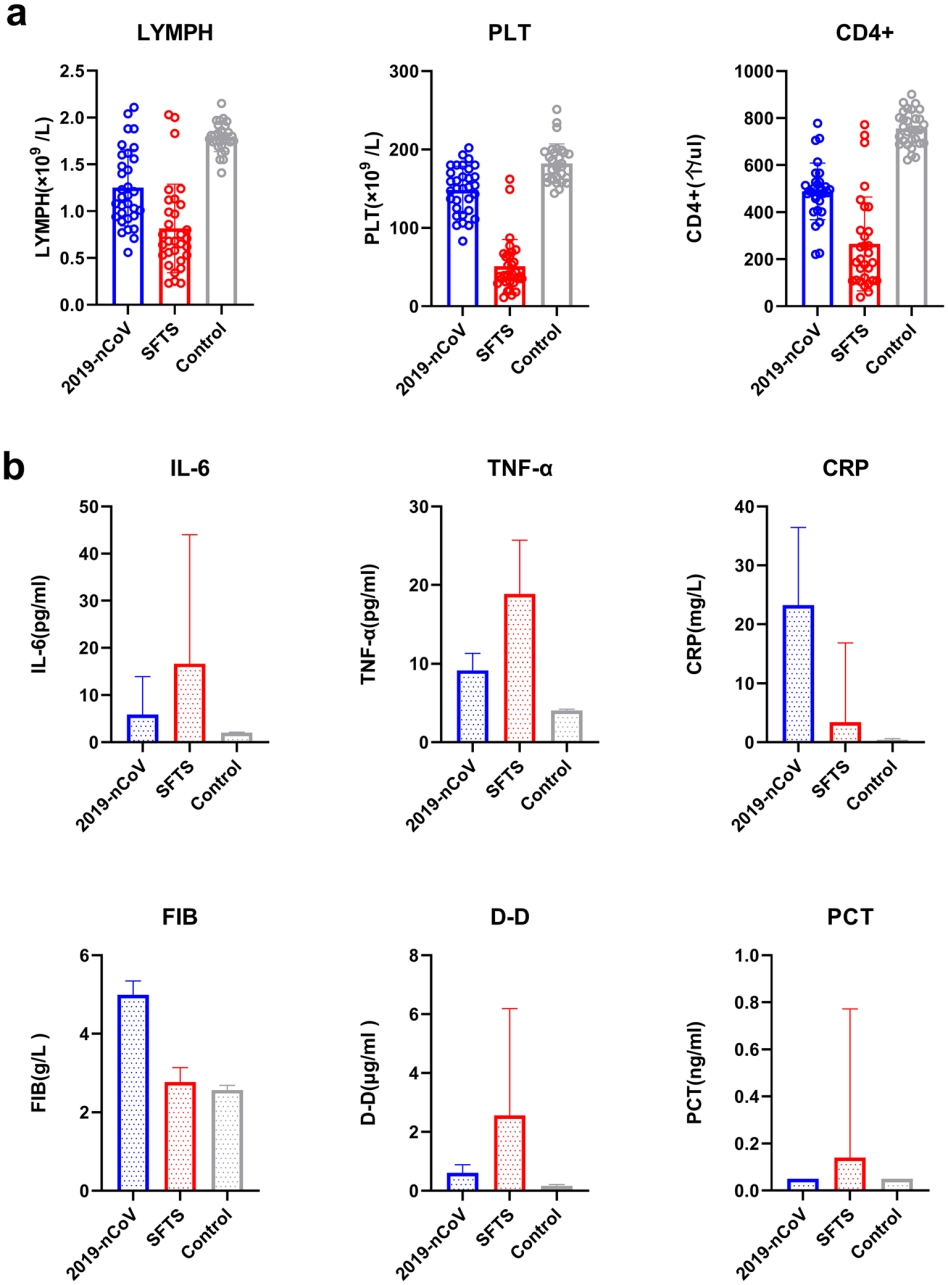
Comparison of indices showing the differences among the 2019-nCoV group, the SFTS group and the control group. (**a**) Comparison of LYMPH, PLT and CD4^+^ T among the 3 groups (statistical identification is represented by “Mean ± SD”). (**b**) Comparison of IL-6, TNF-α, CRP, FIB, D-D and PCT among the 3 groups (statistical identification is represented by “Median with interquartile range”).

A comparison among the 3 groups showed significant differences in WBC count, LYMPH count, PLT count and CD4^+^ T cells count (*P* < 0.05 for all). By pairwise comparison, the WBC count of the control group was significantly higher than that of the 2019-nCoV group and the SFTS group (*P* < 0.05 for both); however, the difference between the 2019-nCoV group and the SFTS group showed no statistical significance (*P* > 0.05). The PLT count of the SFTS group was significantly lower than those of the 2019-nCoV group and the control group (*P* < 0.05 for both). In addition, the PLT count of the 2019-nCoV group was significantly lower than that of the control group (*P* < 0.05). The amounts of LYMPH and CD4^+^ T cells in the SFTS group were significantly lower than those in the 2019-nCoV group and the control group (*P* < 0.05 for both), and those of the 2019-nCoV group were significantly lower than those of the control group (*P* < 0.05 for both). A comparison of the CD8^+^ T cells count and CD4^+^ T/CD8^+^ T ratio among the 3 groups showed no significant differences (*P* > 0.05 for all). Referred to Table 1 and Fig. 1.

**Table 1:** Comparison of WBC, PLT, LYMPH, CD4^+^ T cell count, CD8^+^ T cell count, and CD4^+^/CD8^+^ ratio among the 3 groups (X ± S).

Group	2019-nCoV (n = 32)	SFTS (n = 31)	Control (n = 30)	F	*P*
WBC (× 10^9^/L)	4.21 ± 0.82^#^	3.49 ± 2.42^#^	6.94 ± 1.70	32.104	0.000
LYMPH (× 10^9^/L)	1.25 ± 0.40*^#^	0.82 ± 0.47^#^	1.79 ± 0.15	51.859	0.000
PLT (× 10^9^/L)	148 ± 30*^#^	51 ± 34^#^	182 ± 25	157.221	0.000
CD4^+^ T cells (/µl)	488 ± 156*^#^	265 ± 199^#^	755 ± 73	78.397	0.000
CD8^+^ T cells (/µl)	291 ± 105	223 ± 185	378 ± 32	3.870	0.074
CD4^+^/CD8^+^ ratio	1.77 ± 0.52	1.55 ± 1.08	1.58 ± 0.17	0.582	0.561

Compared with the SFTS group, *P < 0.05; compared with the control group, ^#^P < 0.05.

### Comparison of IL-6, TNF-α, CRP, PCT, FIB and D-D among the 3 groups

A comparison of IL-6, TNF-α, CRP, PCT, FIB and D-D among the 3 groups showed significant differences for all (*P* < 0.05). In the pairwise comparison, the IL-6, TNF-α, PCT and D-D levels of the SFTS group were significantly higher than those of the 2019-nCoV group and the control group (*P* < 0.05 for all). The IL-6, TNF-α and D-D levels of the 2019-nCoV group were also significantly higher than those of the control group (*P* < 0.05 for all), while the PCT of the 2019-nCoV group indicated no significant difference compared with that of the control group (*P* > 0.05). For CRP and FIB, the 2019-nCoV group had significantly higher levels than the SFTS group and the control group (*P* < 0.05 for both), and the SFTS group also had significantly higher levels than the control group (*P* < 0.05). Referred to Table 2 and Fig. 1.

**Table 2:** Comparison of IL-6, TNF-α, CRP, PCT, FIB and D-D among the 3 groups [M (P25–P75)].

Group	2019-nCoV(n = 32)	SFTS(n = 31)	Control (n = 30)	H	*P*
IL-6 (pg/ml)	5.89 (4.36–13.92)*^#^	16.70 (3.44–44)^#^	2.00 (2.00–2.00)	45.101	0.000
TNF-α (pg/ml)	9.15 (8.17–11.27)*^#^	18.9 (15.7–36.45)^#^	4.00 (4.00–4.02)	72.824	0.000
CRP (mg/L)	23.25 (11.31–36.45)*^#^	3.40 (0.70–16.80)^#^	0.30 (0.30–0.40)	59.809	0.000
PCT (ng/ml)	0.05 (0.05–0.05)*	0.14 (0.05–0.77)^#^	0.05 (0.05–0.05)	20.606	0.000
FIB (g/L)	4.99 (4.25–5.34)*^#^	2.77 (2.48–3.13)^#^	2.56 (2.31–2.68)	60.175	0.000
D-D (μg/ml)	0.61 (0.28–0.88)*^#^	2.56 (1.26–6.19)^#^	0.16 (0.12–0.21)	64.149	0.000

Compared with the SFTS group, *P < 0.05; compared with the control group, ^#^P < 0.05.

### Evaluation of the diagnostic efficacy of laboratory indices with significant differences based on a comparison between the 2019-nCoV group and the SFTS group

**Figure 2:**
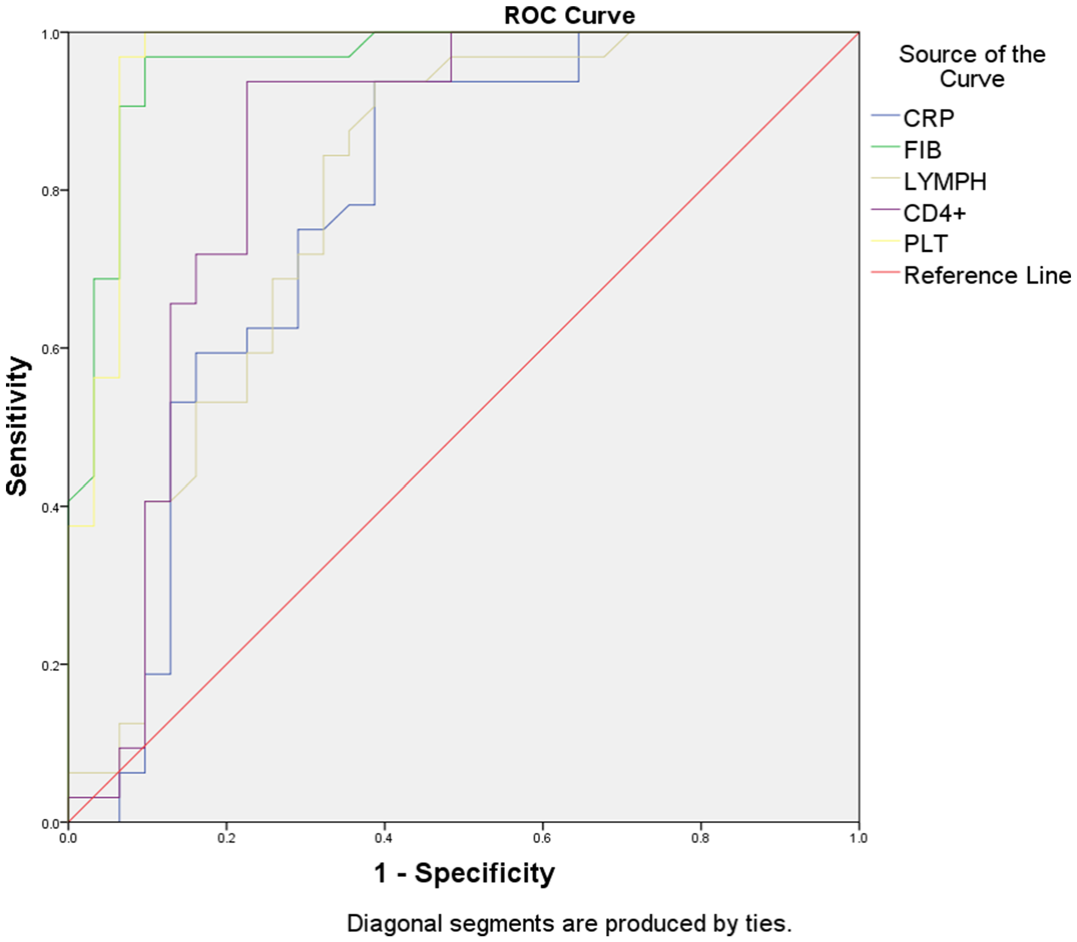
ROC curves for CRP, FIB, LYMPH, PLT and CD4^+^ T cells.

**Figure 3:**
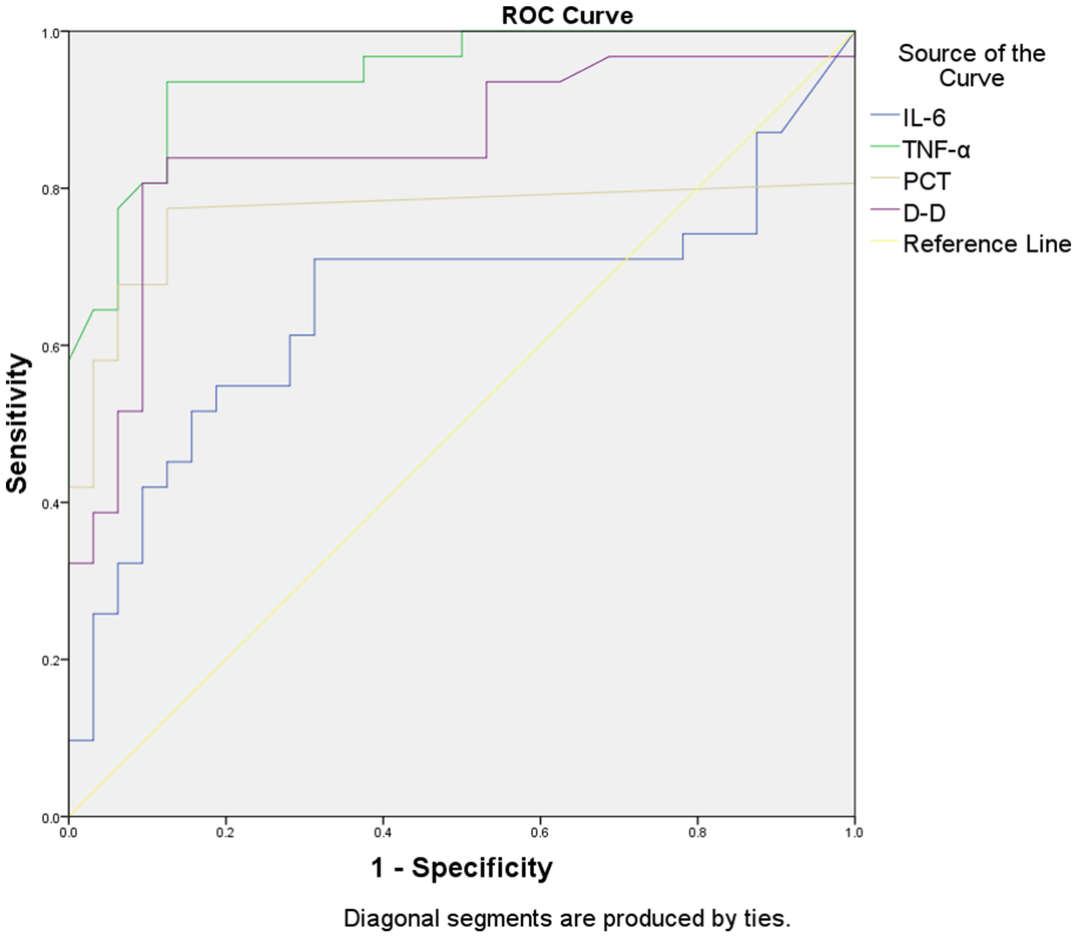
ROC curves for IL-6, TNF-α, PCT and D-D.

ROC curve analysis was performed for CRP, FIB, LYMPH, PLT and CD4^+^ T (the 2019-nCoV group was selected for the outcome variables). Both FIB and PLT showed high diagnostic efficacy with an AUC > 0.85 (refer to Fig. 2 and Table 3). ROC curve analysis was also performed for IL-6, TNF-α, PCT and D-D (because AUC < 0.5, the SFTS group was selected for the outcome variables), where the AUC for TNF-α was > 0.85, showing high diagnostic efficacy (refer to Fig. 3 and Table 4).

**Table 3:** Parameters related to the ROC curves of CRP, FIB, LYMPH, PLT and CD4^+^ T cells.

Indicator	AUC	Cutoff	Sensitivity (%)	Specificity (%)
CRP (mg/L)	0.761	6.15	93.8	63.0
FIB (g/L)	0.955	3.46	96.9	88.9
LYMPH (× 10^9^/L)	0.779	0.76	93.8	59.3
PLT (× 10^9^ /L)	0.959	95	96.9	92.6
CD4^+^ T cells (/μl)	0.826	331	88.9	76.7

The outcome variables are taken from the 2019-nCoV group.

**Table 4:** Parameters related to the ROC curves of IL-6, TNF-α, PCT and D-D.

Indicator	AUC	Cutoff	Sensitivity (%)	Specificity (%)
IL-6 (pg/ml)	0.616	7.67	66.7	68.7
TNF-α (pg/ml)	0.936	13	92.6	87.5
PCT (ng/ml)	0.733	0.051	74.1	87.5
D-D (μg/ml)	0.837	1.22	77.8	90.6

The outcome variables are taken from the SFTS group.

## Discussion

Studies have shown that the infection fatality rate of SARS-CoV-2 in China is 2.5%^8^. Compared to SFTSV infection^13^, SARS-CoV-2 has a lower fatality rate, but a higher infectivity rate. The mean infection fatality rate of SFTS in China is 5.3%^14^. The effects of cell immunodeficiency and increased proinflammatory cytokines on the pathogenesis of both viruses have also been reported^15,17^. In this study, for SARS-CoV-2-infected patients and SFTSV-infected patients, a comparative analysis of laboratory indices, such as cytokines, relative inflammation indices and lymphocyte subsets, was conducted to provide laboratory evidence for early and differential diagnosis of the two infections before collecting positive nucleic acid testing results.

The results of this study showed that, compared with the control group, the WBC counts, absolute LYMPH counts, PLT counts and absolute CD4^+^ T cells counts of the 2019-nCoV group decreased significantly, showing a statistically significant difference (*P* < 0.05), while the absolute counts of IL-6, TNF-α, CRP, FIB and D-D increased significantly, showing a statistically significant difference as well (*P* < 0.05); and the absolute counts of PCT and CD8^+^ T cells and CD4^+^ T/CD8^+^ T showed no statistically significant difference (*P* > 0.05). This corresponds with the descriptions in the *Diagnosis and Treatment Plan for Novel Coronavirus Pneumonia* (amendment of the trial Eighth Edition)^16^; on the basis of relative studies^6–9^, excessive inflammation and the "cytokine storm" exist in the SARS-CoV-2 infection process, and a large amount of IL-6, TNF-α and other cytokines are released, stimulating liver cells to produce acute phase proteins (APPs), such as CRP and FIB. Meanwhile, a large number of cytokines can damage vascular endothelial cells, causing abnormal coagulation and increased D-D levels^18^. To maintain a stable internal environment, the body restrains immunocyte functions by inducing the apoptosis of immune cells (such as CD4^+^ T cells) to resist the effects of inflammatory cytokines. This leads to an immunosuppressive state ^19^. Relevant studies have shown that in the virus infection process^20^, the PLT count decreases to a certain extent. Therefore, the results of this study are consistent with relevant research.

Compared with the 2019-nCoV group, the WBC count, absolute LYMPH count, PLT count and absolute CD4^+^ T cells count of the SFTS group were much lower, showing statistical significance (*P* < 0.05). This indicates that the influence of SFTSV infection on cell immunodeficiency is higher than that of SARS-CoV-2 infection. Previous studies have shown^21^ that SFTSV promotes macrophage phagocytosis of platelets by sticking to the platelets, leading to a significant reduction. This is consistent with the results of this study. The levels of IL-6, TNF-α, D-D and PCT in the SFTS group were significantly higher than those in the 2019-nCoV group (*P* < 0.05). This is consistent with the fact that SFTS infection features a high lethal rate and a high rate of severe cases^13^. Compared with the SFTS group, the levels of CRP and FIB of the 2019-nCoV group were higher, showing a statistically significant difference (*P* < 0.05). As acute phase response proteins, CRP and FIB may increase in many diseases, such as infection and autoimmune injury. With regard to the different rising levels in the two patient groups, the author believes that it may be related to the release of relative inflammatory mediators.

## Conclusions

In conclusion, this study indicates that at the initial stage of SARS-CoV-2 and SFTS infection, the blood indices of patients are distinct from each other, including WBC count, absolute LYMPH count, PLT count, absolute CD4^+^ T cells count, and IL-6, TNF-α, D-D, CRP and FIB levels. In addition, PLT, FIB and TNF-α are of high diagnostic value. Because their AUCs are greater than 0.85, these indices may be measured as an effective supplement to nucleic acid testing. The results of this study provide certain laboratory evidence for the early and differential diagnosis of SARS-CoV-2 and SFTSV infection.
